# Supplementary material for: Ochratoxin A Defective Aspergillus carbonarius Mutants as Potential Biocontrol Agents
Source: Toxins (Basel). 2022 Oct 31;14(11):745. doi: 10.3390/toxins14110745 (PMC9695793; doi:10.3390/toxins14110745)
Supplement: Supplementary file 1 [file toxins-14-00745-s001.zip › toxins-1978247-supplementary.pdf]

A

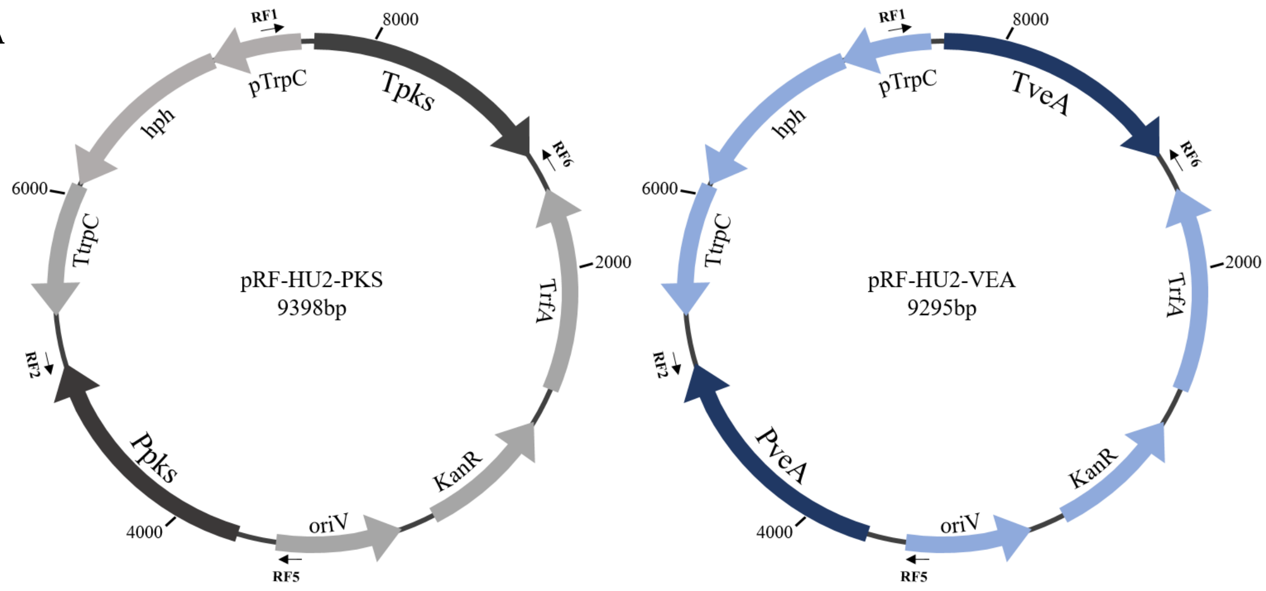
RF1
8000
pTrpC
Tpks
RF6
hph
6000
TtrpC
pRF-HU2-PKS
9398bp
2000
TrfA
RF2
Ppks
KanR
oriV
4000
RF5
RF1
8000
pTrpC
TveA
RF6
hph
6000
TtrpC
pRF-HU2-VEA
9295bp
2000
TrfA
RF2
PveA
KanR
oriV
4000
RF5

B

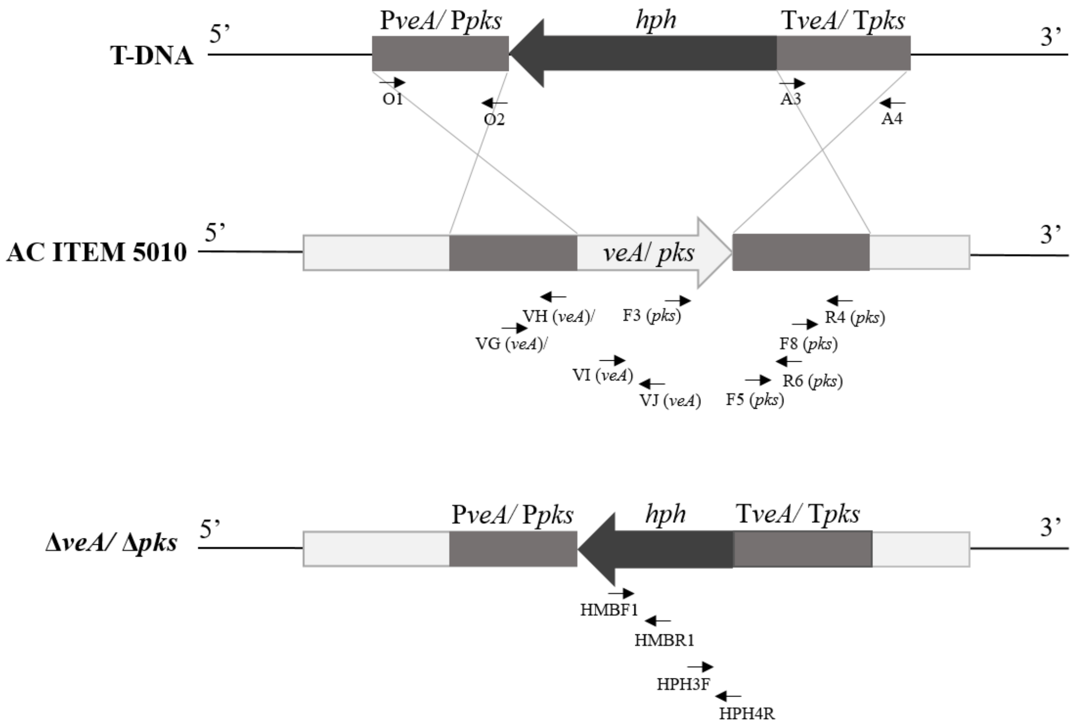
T-DNA
5'
PveA/ Ppks
hph
TveA/ Tpks
3'
O1
O2
A3
A4
AC ITEM 5010
5'
veA/ pks
3'
VH (veA)/
F3 (pks)
R4 (pks)
VG (veA)/
F8 (pks)
VI (veA)
R6 (pks)
VJ (veA)
F5 (pks)
ΔveA/ Δpks
5'
PveA/ Ppks
hph
TveA/ Tpks
3'
HMBF1
HMBR1
HPH3F
HPH4R

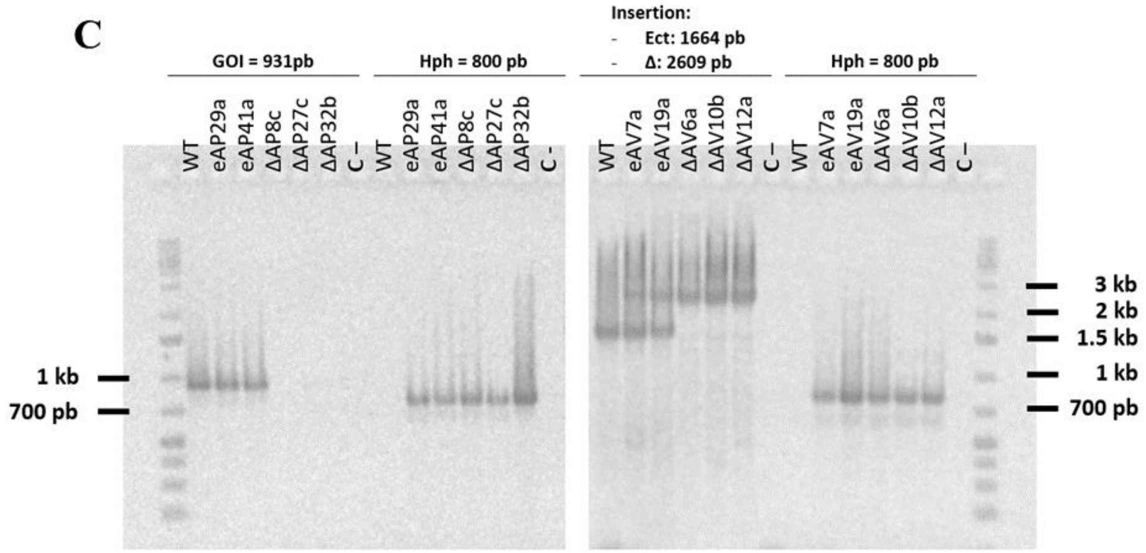

Figure S1. Deletion of the *pks* and *veA* genes in *Aspergillus carbonarius*. (A) Physical maps of plasmids pRFHU2-PKS and pRFHU2-VEA. (B) Diagram of the deletion cassette used to replace the target region (gene of interest, GOI) in the wild-type strain by the hygromycin resistant marker (Hph) by homologous recombination, generating Δ*pks* and Δ*veA* mutants. Primers used in the construction and the analysis of both plasmids are shown. (C) Amplification band patterns of the different polymerase chain reactions (PCR): gene of interest (GOI) analysis of the mutants in the *otaA* (*pks*) and *veA* gene in the left and right panels, respectively. Analyses were performed for wild-type (wt) ITEM 5010, two ectopic mutants (preceded by the letter "e") and three knockout mutants (preceded by the symbol "Δ").

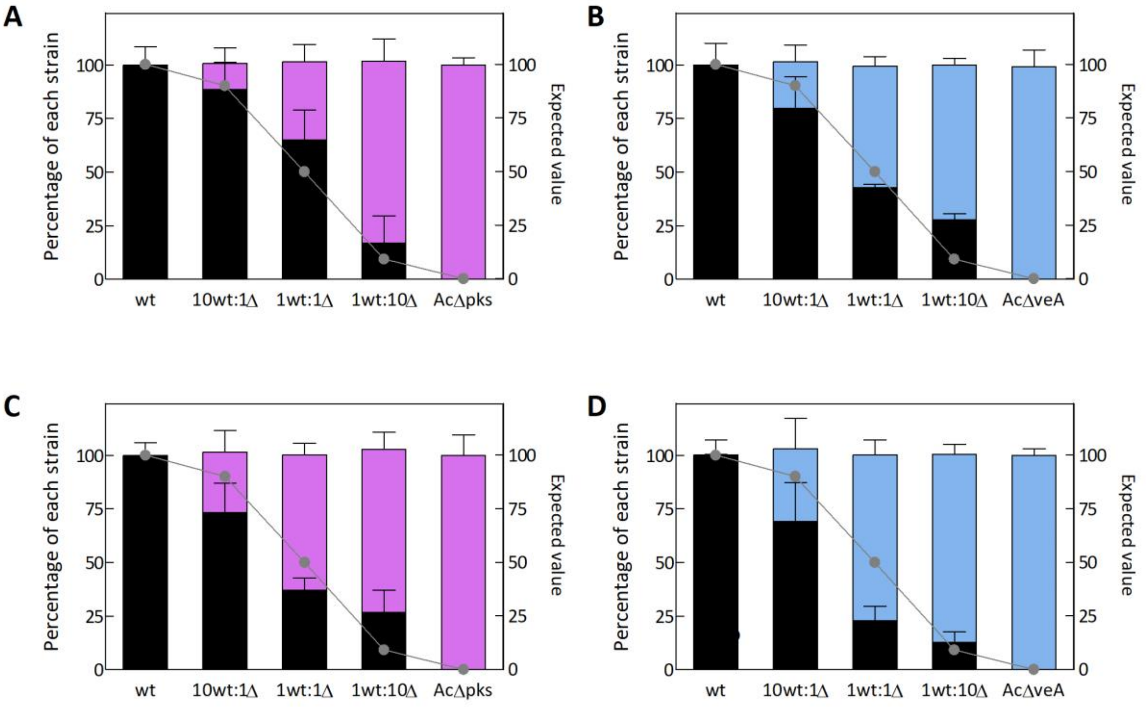

Figure S2. Competitiveness of Δ*pks* (A-C, pink bars) and Δ*veA* (B-D, blue bars) knockout mutants against the mycotoxigenic wild-type strain *A. carbonarius* ITEM 5010 (black bars) at day 0 (A-B) and 7 days' post inoculation (C-D). Competitiveness was determined by counting colonies on PDA and PDA supplemented with 100 ug/mL of hygromycin. Only knockout mutants are able to grow on PDA supplemented with the antibiotic. Values are the mean of at least three biological replicates and error bars represent the standard error of the mean (SEM).

Table S1. Estimation of the number of T-DNA copies that have been integrated into the genome of the mutants.

Strain	Genotype	Cq goi	Cq NRPS	Estimated T-DNA copy number
ITEM 5010	Wild type	23.41 ± 0.29	21.37 ± 0.05	-
Epks29a	Ectopic	22.37 ± 0.01	21.15 ± 0.26	1
Epks41a	Ectopic	22.24 ± 0.14	21.16 ± 0.09	1
Δpks8a	knockout	23.27 ± 0.30	21.29 ± 0.08	1
Δpks27c	knockout	23.87 ± 0.14	21.46 ± 0.12	1
Δpks32b	knockout	23.75 ± 0.11	21.04 ± 0.24	1
ITEM 5010	Wild type	22.90 ± 0.36	22.85 ± 0.29	-
EveA7a	Ectopic	21.68 ±0.44	23.11 ± 0.62	1
EveA9a	Ectopic	19.34 ± 0.10	22.11 ± 0.12	4
ΔveA6a	knockout	23.23 ± 0.16	23.51 ± 0.07	1
ΔveA10b	knockout	21.89 ± 0.24	21.97 ± 0.05	1
ΔveA12a	knockout	22.10 ± 0.23	22.10 ± 0.27	1

Table S2. List of primers used in this study.

Name	Sequence (5′ → 3′)	Description
**Δ*pks* mutant**		
OTApks_O1	**GGTCTTAAU**GCTCTATATCGCGCGCAAAG	Amplification of upstream region
OTApks_O2	**GGCATTAAU**GGCCCGGCTTCTTAAGACTT	Amplification of upstream region
OTApks_A3	**GGACTTAAU**CCGCCCTCTCGAGTGTAAAG	Amplification of downstream region
OTApks_A4	**GGGTTTAAU**GGGTTGGTCTTTGGCGTAGA	Amplification of downstream region
OTApks-F3	GAACCATTTCCGACCTTCT	Screening gene deletion
OTApks-R4	TTCGAGGATGGCAAGTAGA	Screening gene deletion, T-DNA copy number
OTApks-F5	AGAAGTGTAGTGCGCTGGATG	PCR (growth)
OTApks-R6	ATCTGCACAGAGTTGCTTGG	PCR (growth)
OTApks-F8	CAACTATTCGGCAGCCAACG	T-DNA copy number
**Δ*veA* mutant**		
veA-VA-O1	**GGTCTTAAU**CCGAGGTCCAAAGATGGCAGGAC	Amplification of upstream region[1]
veA-VA-O2	**GGCATTAAU**ACATTGCATGCGGGGGATCGATG	Amplification of upstream region[1]
veA-VA-A3	**GGACTTAAU**CAGATTGACCCGACTCCGAACGAC	Amplification of downstream region[1]
veA-VA-A4	**GGGTTTAAU**TGGTCCAGCTCTTCGGCGTCATC	Amplification of downstream region[1]
veA-VI	TCCCGGTTCTCACAGGCGTA	Screening gene deletion[1]
veA-VJ	GCTGTCCTTGGTCTCCTCGTA	Screening gene deletion[1]
veA-VG	GAGTACACCGGCTGGTGGTTAGGA	T-DNA copy number[1]
veA-VH	TCTTTCCATCGCGGGTGATTCGGCT	T-DNA copy number[1]
**Other primers**		
RF-1	AAATTTTGTGCTCACCGCCTGGAC	*E. coli* colonies selection and DNA sequencing
RF-2	TCTCCTTGCATGCACCATTCCTTG	*E. coli* colonies selection and DNA sequencing
RF-5	GTTTGCAGGGCCATAGAC	*E. coli* colonies selection and DNA sequencing
RF-6	ACGCCAGGGTTTTCCCAGTC	*E. coli* colonies selection and DNA sequencing
HMBF1	CTGTCGAGAAGTTTCTGATCG	Screening insertion resistant marker
HMBR1	CTGATAGAGTTGGTCAAGACC	Screening insertion resistant marker
HPH3F	TATGTCCTGCGGGTAAATAGCTG	PCR (growth)
HPH4R	GAGATGCAATAGGTCAGGCTCTC	PCR (growth)
AcNRP_F	CTCCACCCATCCTCCCGTTC	*nrps* as reference gene [1]
AcNRP_R	AATCCATGTCCTCACCATCGC	*nrps* as reference gene [1]

[1] Crespo-Sempere, A.; Marín, S.; Sanchis, V.; Ramos, A.J. VeA and LaeA Transcriptional Factors Regulate Ochratoxin A Biosynthesis in *Aspergillus Carbonarius*. Int. J. Food Microbiol. 2013, 166, 479–486, doi:10.1016/j.ijfoodmicro.2013.07.027.
